# The influence of rural residency on the uptake of screening for breast, cervical, and colorectal cancers in Scotland

**DOI:** 10.1093/pubmed/fdag018

**Published:** 2026-02-27

**Authors:** Lisa Iversen, Edwin Amalraj Raja, Mintu Nath, Gerald Lip, Valerie Speirs, Jamie Collins, Lorna Philip, Sharon J B Hanley, Peter Murchie

**Affiliations:** Academic Primary Care, Institute of Applied Health Sciences, University of Aberdeen, Polwarth Building, Foresterhill, Aberdeen AB25 2ZD, UK; Biostatistics & Health Data Science, Institute of Applied Health Sciences, University of Aberdeen, Polwarth Building, Foresterhill, Aberdeen AB25 2ZD, UK; Biostatistics & Health Data Science, Institute of Applied Health Sciences, University of Aberdeen, Polwarth Building, Foresterhill, Aberdeen AB25 2ZD, UK; North East Scotland Breast Screening Programme, NHS Grampian, Aberdeen Royal Infirmary, Foresterhill Road, Aberdeen AB25 2XF, UK; Institute of Medical Sciences, University of Aberdeen, Foresterhill, Aberdeen AB25 2ZD, UK; NHS Grampian, Aberdeen Royal Infirmary, Foresterhill, Aberdeen AB25 2ZN, UK; Geography & Environment, School of Geosciences, University of Aberdeen, King's College, Aberdeen AB24 3UE, UK; Academic Primary Care, Institute of Applied Health Sciences, University of Aberdeen, Polwarth Building, Foresterhill, Aberdeen AB25 2ZD, UK; Academic Primary Care, Institute of Applied Health Sciences, University of Aberdeen, Polwarth Building, Foresterhill, Aberdeen AB25 2ZD, UK

**Keywords:** adults, cancer, screening

## Abstract

**Background:**

There has been little examination of rural–urban residency and cancer screening. We investigated rural–urban variations in breast, cervical, and colorectal cancer screening uptake across Scotland.

**Methods:**

Using aggregate data and cross-sectional analyses, we calculated uptake and detection rates (breast and bowel screening). We modelled uptake by urban and rural residency, age group, sex (bowel screening only), year, health board, and deprivation using multivariable logistic regression with appropriate interaction terms.

**Results:**

Cervical screening uptake was higher in rural than urban areas (under 50s: 75.9% vs. 69.1%; 50+: 77.5% vs. 75.3%). Mammography uptake was: 77.0% in rural; 71.0% in urban areas. Bowel screening uptake was: 56.6% in urban; 62.5% in rural areas. In multivariable models, two-way interaction effects between residency and deprivation, and between residency and health board, were statistically significant (*P* < .05). Rural residency did not confer universally higher uptake. Breast and bowel cancer detection were similar in both areas.

**Conclusions:**

The relationship between residence and cancer screening uptake varied across Scotland. Uptake patterns were complex and not consistent across all rural areas, likely reflecting Scotland’s topography and characteristics of the screening eligible. Efforts to improve screening uptake would likely benefit from local rather than national public health policies.

## Introduction

Worldwide rural residency is associated with poorer cancer survival.^1^ Reasons for this are not well understood, with research focused upon different cancer pathway milestones. For instance, studies have reported rural dwellers are more likely than urban dwellers to be diagnosed with later stage cancer,^2–5^ suggesting that rural patients have delays in presenting symptoms, or in subsequent investigations. Poorer cancer survival in rural dwellers has been suggested as due to differences in: socioeconomic circumstances influencing how rural and urban patients use health services^6^ and, cancer treatment and follow-up care, stemming from rural patients travelling significant distances for diagnostic care and treatment.^7–10^

Cervical,^11^ breast,^12^ and bowel screening^13^ programmes are all associated with reduced cancer mortality. There has been less examination of whether there is a lower uptake of cancer screening among rural dwellers in comparison to those who reside in urban areas. If screening uptake is demonstrated to be lower in rural compared to urban communities, it seems plausible that a lower proportion of early stage, potentially curable cancers are being identified there, which could contribute to rural cancer inequality.

A previous systematic review of the influence of rural residence on cancer screening uptake found generally lower uptake of breast, cervical, and colorectal cancer screening among rural residents, but few studies were from countries with universally free of charge programmes.^14^ Colorectal cancer screening uptake was lower in rural areas (odds ratio, OR 0.66, 95% confidence interval, CI 0.50 to 0.87, I^2^ = 85%), but breast cancer screening uptake was similar in rural and urban locations (OR 0.93, 95% CI 0.80 to 1.09, I^2^ = 86%). However, these meta-analyses were limited by high heterogeneity, and only one eligible study prohibited meta-analysis for cervical screening.

In Scotland, 14 regional National Health Service (NHS) health boards are responsible for delivering health care, including cancer screening. According to the Scottish Government’s Urban Rural Classification, ~95% of the land mass is rural, but only 21% of Scotland’s population live rurally.^15^ Scotland’s population is geographically concentrated in the towns and cities of the Central Belt, the area between the Firths of Clyde and Forth, with a further concentration residing along the east coast. Northern and southern Scotland is predominantly rural, with the northwest Highlands containing the majority of the United Kingdom’s (UK’s) mountainous terrain. The Highland local authority area covering around a third of Scotland’s land mass, is the UK’s least populated region (8 persons/km^2^).^16^ Over 750 islands, of which 93 are inhabited, form five geographical groupings; the Shetland Isles, the Orkney Isles, inner and outer Hebrides (the latter known as the Western Isles), and the Clyde Islands. Some health boards serve predominantly urban or rural-dwelling populations and some mixed urban and rural populations. NHS Scotland provides population-based breast, cervical, and colorectal cancer screening programmes free of charge to patients registered with a general practitioner (GP). To date, the impact of rural residence on cancer screening uptake in Scotland has not been explored. Using country-wide data, we investigated whether there are rural–urban variations in cervical, breast, and bowel cancer screening uptake across Scotland.

## Methods

### Population

Since 1988, the cervical screening programme has offered triennial screening for women aged 20–60 years,^17^ supported since 2007 by a national IT system, the Scottish Cervical Call Recall System. From June 2016 women aged 25–49 years were offered screening triennially and those aged 50–64 years plus 364 days every 5 years. In March 2020 human papillomavirus testing became the primary screening method with a 5-year interval. Those contacted by the Scottish Cervical Call Recall System usually make a screening appointment at their general practice.

The Scottish Breast Screening Programme commenced in 1988 with full coverage by 1991.^18^ Women aged 50–64 years were invited for triennial mammography until 2003–2004 when the age range became 50–70 years plus 364 days. The screening programme is underpinned by a national IT system, the Scottish Breast Screening System. Depending on home address, women travel to one of six breast screening centres (located in large urban centres) or to one of 20 travelling mobile units operated by the centres to provide widespread geographical screening coverage.^19^

After piloting, the Scottish Bowel Screening Programme started in June 2007 with full coverage by December 2009.^20^ Faecal immunochemical test (FIT) kits are mailed biennially to everyone aged 50–74. Participants use the FIT kit to collect a sample at home which they return by mail. FIT replaced the faecal occult blood test (FOBT) in November 2017.

### Measures

Aggregate cancer screening data (individual-level data were not available for research) were obtained from Public Health Scotland (approval reference DP2122–0245 IR2021–00460) for cross-sectional analyses. Due to age-dependent screening intervals, there were two datasets (age group 25–49 and 50–64 years) derived from the Scottish Cervical Call Recall System. For years 2016/17 to 2019/20 (screening year was from 1^st^ April to 31^st^ March), the datasets contained the number eligible for cervical screening, and the total number screened by: age group (5-year groups); health board; Scottish Index of Multiple Deprivation (SIMD) quintile^21^; and Scottish Government Urban Rural Classification (6-fold version).^22^

The breast screening dataset was extracted from the Scottish Breast Screening System and included for the years 2016/17 to 2019/20, the number: invited for and attended screening; of invasive cancers detected by: age group (5-year groups); health board, SIMD quintile and Scottish Government Urban Rural Classification (6-fold version).

The data from the Scottish Bowel Screening Programme, from 2009/10 to 2020/21, comprised the number: invited, screened and invasive cancers detected by: age group (5-year groups), sex, health board, SIMD quintile, and the Scottish Government Urban Rural Classification (6-fold version).

The Scottish Government Urban Rural Classification is an area-based typology using population size and accessibility (based on drive time) as the basis of 2-, 3-, 6-, and 8-fold territorial classifications.^22^ To allow comparison of screening uptake in rural and urban areas, using the Classification guidance, we used the ‘core’ rural definition, where the 6-fold classification was collapsed into the 2-fold version. Areas with populations less than 3000 are classified as rural (categories 5–6), and categories 1–4 (all with populations ≥3000) are classified as ‘rest of Scotland’ which we term urban.

### Statistical analysis

For each screening dataset, we estimated the screening uptake rate among those invited. For the breast and bowel screening datasets, we estimated the detection rate. To evaluate the association of uptake with potential covariates, we fitted multivariable logistic regression models on the grouped data with the corresponding number of invitations as the binomial total. The model incorporated residency (urban and rural), age group, sex (bowel cancer screening only), screening year, health board (14 boards) and SIMD quintile as predictors. We also investigated two-way interaction effects between residential status (rural/urban) and SIMD quintile, and between residential status and health board. If statistically significant, the two-way interaction effect was retained in the model. The model accounted for the overdispersion in the data, and the standard errors of estimates were obtained using the robust ‘sandwich’ variance estimator. We estimated unadjusted odds ratios (OR) and 95% CI from the single variable model and the adjusted odds ratios (aOR) with 95% CI for all relevant predictors from the multivariable model.

We also conducted a sensitivity analysis, using analogous multivariable logistic regression models, to examine the association with drive time to settlements of at least 10 000 people as a predictor, replacing residency (based on population size). We obtained the drive time groups from the 6-fold Scottish Government Urban Rural Classification: combining categories 1, 2, 3, and 5 (urban areas and areas within a 30-minute drive time to settlements ≥10 000 people), and combining categories 4 and 6 (over 30-minute drive time to settlements ≥10 000 people). Data from island health boards (Orkney, Shetland, and Western Isles) were excluded from the sensitivity analysis due to limited information. All analyses were conducted using STATA version 17 MP and R version 4.4. A p-value < .05 was considered statistically significant.

## Results

### Cervical screening

Among under 50s, cervical screening uptake was higher in rural (75.9%) than urban (69.1%) areas (Table 1). Uptake was lowest in the youngest (61.3% in patients aged 25–29 years) and among those living in the most deprived SIMD quintile (65.5%). Although uptake was higher than 70% in most (12/14) health boards, there were geographical variations. After adjustment, the odds of uptake were 1.77 (95% CI 1.73–1.82) times higher in the 45–49 compared to the 25–29 age group. For those living in the most deprived SIMD quintile, there was no difference in screening uptake by residency. Uptake was higher in rural than urban areas in all other quintiles. The association between residency and screening uptake varied by health board. Four health boards had higher uptake from rural vs. urban residents (Grampian, aOR 1.43 (95% confidence interval, CI 1.34–1.53), Greater Glasgow and Clyde, aOR 1.58 (95% CI 1.37–1.82), Lothian, aOR 1.66 (95% CI 1.52–1.82), and Shetland, aOR 1.56 (95% CI 1.16–2.11). There were no rural vs. urban differences elsewhere.

**Table 1:** Cervical screening uptake (screening interval 3.5 years) during 2016 to 2020 among women aged 25–49 in Scotland.

Group	Screened/Invited, N	Uptake %	Unadjusted odds ratio (95% CI)	Adjusted odds ratio (95% CI)^a^
Residency				
Urban	2 110 704/3 056 455	69.1	1.00	-
Rural	363 797/479 118	75.9	**1.41 (1.36–1.47)**	-
Year				
2016/17	628 224/888 092	70.7	1.00	1.00
2017/18	621 854/884 877	70.3	0.98 (0.90–1.06)	0.98 (0.96–1.00)
2018/19	622 604/881 987	70.6	0.99 (0.91–1.08)	1.00 (0.98–1.02)
2019/20	601 819/880 617	68.3	**0.89 (0.82–0.97)**	**0.90 (0.88–0.92)**
Age group, years				
25–29	471 391/769 403	61.3	1.00	1.00
30–34	490 705/715 357	68.6	**1.38 (1.28–1.49)**	**1.38 (1.35–1.41)**
35–39	499 760/692 514	72.2	**1.64 (1.52–1.77)**	**1.60 (1.57–1.64)**
40–44	472 828/636 558	74.3	**1.83 (1.69–1.97)**	**1.75 (1.71–1.79)**
45–49	539 817/721 741	74.8	**1.88 (1.74–2.03)**	**1.77 (1.73–1.82)**
SIMD quintile				Residency^*^SIMD
1 (most deprived)	516 872/788 823	65.5	**0.72 (0.64–0.81)**	1.08 (0.97–1.21)
2	505 898/736 485	68.7	**0.83 (0.73–0.95)**	**1.16 (1.08–1.25)**
3	474 132/674 113	70.3	**0.90 (0.79–1.03)**	**1.19 (1.13–1.26)**
4	492 975/667 644	73.8	1.07 (0.94–1.22)	**1.11 (1.05–1.17)**
5 (least deprived)	484 624/668 508	72.5	1.00	**1.23 (1.12–1.35)**
Health Board (HB)				Residency^*^HB
Ayrshire & Arran	160 021/217 686	73.5	**1.44 (1.33–1.55)**	1.03 (0.94–1.12)
Borders	45 268/60 592	74.7	**1.53 (1.42–1.66)**	0.88 (0.78–1.00)
Dumfries & Galloway	60 225/79 236	76.0	**1.64 (1.53–1.76)**	0.95 (0.85–1.07)
Fife	156 358/219 365	71.3	**1.29 (1.19–1.39)**	1.04 (0.95–1.14)
Forth Valley	140 533/188 966	74.4	**1.50 (1.39–1.63)**	1.11 (1.00–1.22)
Grampian	258 956/360 241	71.9	**1.33 (1.22–1.45)**	**1.43 (1.34–1.53)**
Greater Glasgow & Clyde	566 185/859 851	65.8	1.00	**1.58 (1.37–1.82)**
Highland	130 690/179 332	72.9	**1.39 (1.30–1.50)**	1.06 (0.99–1.15)
Lanarkshire	316 642/430 219	73.6	**1.45 (1.34–1.56)**	1.05 (0.97–1.15)
Lothian	432 669/654 290	66.1	1.01 (0.91–1.13)	**1.66 (1.52–1.82)**
Orkney	9059/11 570	78.3	**1.87 (1.72–2.04)**	0.94 (0.69–1.27)
Shetland	10 046/12 736	78.9	**1.94 (1.74–2.15)**	**1.56 (1.16–2.11)**
Tayside	178 020/247 906	71.8	**1.32 (1.22–1.43)**	1.07 (0.98–1.15)
Western Isles	9829/13 583	72.4	**1.36 (1.25–1.48)**	1.13 (0.86–1.48)

^a^Adjusted for year, age group and the interactions residency^*^SIMD and residency^*^Health Board. For residency^*^SIMD, estimates of the adjusted odds ratio (95% CI) represent the adjusted odds ratio of rural versus urban residency for each SIMD quintile. For residency^*^Health Board, estimates of the adjusted odds ratio (95% CI) represent the adjusted odds ratio (95% CI) of rural versus urban residency for each Health Board. Estimates in bold denote *P* < .05.

Among those older with a 5.5-year cervical screening interval, uptake was higher in rural areas, 77.5% vs. 75.3% (Table 2). Uptake was highest among the youngest (50–54 years 79.2%) and those living in the least deprived SIMD quintile (82.3%). Uptake varied across health boards. The multivariable model found lower odds of uptake among those older compared with the 50–54 years group. There was an interaction between residency and SIMD; in quintiles 1 (most deprived group) and 2, rural areas had higher odds of uptake, but in quintile 5 (least deprived group) rural areas had lower odds of uptake compared to urban areas. The interaction between residency and health board showed lower odds of uptake in rural compared to urban areas in two health boards (Borders, aOR 0.83, 95% CI 0.79–0.87 and Orkney, aOR 0.68, 95% CI 0.59–0.78), higher in rural areas in five with no differences in seven health boards.

**Table 2:** Cervical screening uptake (screening interval 5.5 years) among women aged 50–64 in Scotland.

Group	Screened/Invited, N	Uptake %	Unadjusted odds ratio (95% CI)	Adjusted odds ratio (95% CI)^a^
Residency				
Urban	1 262 992/1 677 506	75.3	1.00	-
Rural	313 835/404 836	77.5	1.13 (1.09–1.18)	-
Year				
2016/17	387 876/508 064	76.3	1.00	1.00
2017/18	391 932/517 537	75.7	0.97 (0.90–1.04)	**0.97 (0.96–0.98)**
2018/19	398 740/525 190	75.9	0.98 (0.91–1.05)	**0.98 (0.97–0.99)**
2019/20	392 827/531 551	74.9	**0.93 (0.86–0.99)**	**0.93 (0.92–0.94)**
Age group, years				
50–54	606 235/765 724	79.2	1.00	1.00
55–59	542 658/716 582	75.7	**0.82 (0.77–0.87)**	**0.82 (0.81–0.82)**
60–64	427 934/600 036	71.3	**0.65 (0.62–0.69)**	**0.64 (0.64–0.65)**
SIMD quintile				Residency^*^SIMD
1 (most deprived)	262 296/389 826	67.3	**0.44 (0.42–0.47)**	**1.10 (1.05–1.15)**
2	292 998/403 799	72.6	**0.57 (0.55–0.59)**	**1.04 (1.01–1.07)**
3	325 525/428 053	76.0	**0.68 (0.66–0.71)**	1.01 (0.98–1.03)
4	342 758/431 240	79.5	**0.84 (0.80–0.87)**	0.98 (0.95–1.00)
5 (least deprived)	353 250/429 424	82.3	1.00	**0.90 (0.87–0.94)**
Health Board (HB)				Residency^*^HB
Ayrshire & Arran	112 742/151 966	74.2	1.01 (0.92–1.12)	0.96 (0.93–1.00)
Borders	38 182/49 006	77.9	**1.24 (1.14–1.36)**	**0.83 (0.79–0.87)**
Dumfries & Galloway	47 603/62 317	76.4	**1.14 (1.04–1.24)**	0.98 (0.93–1.03)
Fife	107 446/143 883	74.7	1.04 (0.94–1.14)	1.03 (0.99–1.07)
Forth Valley	91 596/119 072	76.9	**1.17 (1.06–1.30)**	1.04 (0.99–1.08)
Grampian	161 941/207 348	78.1	**1.26 (1.15–1.37)**	**1.12 (1.09–1.16)**
Greater Glasgow & Clyde	328 582/444 264	74.0	1.00	1.06 (1.00–1.12)
Highland	100 692/133 328	75.5	1.09 (0.99–1.19)	1.01 (0.98–1.05)
Lanarkshire	198 908/268 638	74.0	1.00 (0.91–1.11)	**1.06 (1.02–1.10)**
Lothian	243 658/314 238	77.5	**1.22 (1.10–1.34)**	**1.12 (1.08–1.17)**
Orkney	6921/8914	77.6	**1.22 (1.08–1.38)**	**0.68 (0.59–0.78)**
Shetland	6778/8505	79.7	**1.38 (1.23–1.55)**	**1.17 (1.02–1.35)**
Tayside	123 989/160 271	77.4	**1.20 (1.10–1.32)**	**1.05 (1.02–1.09)**
Western Isles	7789/10 592	73.5	0.98 (0.88–1.09)	1.04 (0.92–1.17)

^a^Adjusted for year, age group and the interactions residency^*^SIMD and residency^*^Health Board. For residency^*^SIMD, estimates of the adjusted odds ratio (95% CI) represent the adjusted odds ratio of rural versus urban residency for each SIMD quintile. For residency^*^Health Board, estimates of the adjusted odds ratio (95% CI) represent the adjusted odds ratio (95% CI) of rural versus urban residency for each Health Board. Estimates in bold denote *P* < .05.

### Breast screening

Mammography uptake was 77.0% in rural and 71.0% in urban areas (Table 3). The lowest uptake was in the youngest (aged 50–54 years:70.5%) and those living in the most deprived areas (SIMD quintile 1: 59.8%). Uptake varied across health boards (66.4%–84.0%). After adjustment, the odds of uptake were 14% (95% CI 12%, 15%) and 13% (95% CI 11%, 15%) higher in those aged 60–64 and 65–69 than in the 50–54 years group. There were interactions between residency and SIMD, and health board. For those living in SIMD quintiles 1 and 2, the odds of attendance were higher in rural than urban areas. The pattern was different in the least deprived group (SIMD 5) with lower odds of uptake in rural than urban areas (aOR 0.88, 95% CI 0.83–0.93). In three health board areas, the odds of uptake were higher (between 11% and 25%) in rural areas. In two health boards, the odds of uptake were lower (8% and 24%) in rural than urban areas, with no uptake variations in nine health boards. The breast cancer screening programme detected 4242 and 1068 invasive cancers among urban and rural dwellers respectively, demonstrating similar cancer detection rates: urban: 7.25 cancers detected per 1000 screened (95% CI 7.04–7.47); rural: 7.01 (95% CI 6.59–7.42).

**Table 3:** Breast screening uptake among women aged 50–69 in Scotland.

Group	Screened/Invited, N	Uptake %	Unadjusted odds ratio (95% CI)	Adjusted odds ratio (95% CI)^a^
Residency				
Urban	584 678/823 162	71.0	1.00	-
Rural	152 435/197 839	77.0	**1.37 (1.31–1.43)**	-
Year				
2016/17	179 060/248 350	72.1	1.00	1.00
2017/18	179 740/252 266	71.2	0.96 (0.88–1.04)	**0.94 (0.93–0.96)**
2018/19	185 436/252 509	73.4	1.07 (0.97–1.18)	**1.13 (1.11–1.15)**
2019/20	192 877/267 876	72.0	1.00 (0.91–1.09)	1.01 (0.99–1.02)
Age group, years				
50–54	199 001/282 365	70.5	1.00	1.00
55–59	189 504/263 707	71.9	1.07 (0.98–1.17)	**1.07 (1.05–1.08)**
60–64	166 953/227 688	73.3	**1.15 (1.05–1.26)**	**1.14 (1.12–1.15)**
65–69	181 655/247 241	73.5	**1.16 (1.06–1.26)**	**1.13 (1.11–1.15)**
SIMD quintile				Residency^*^SIMD
1 (most deprived)	112 733/188 633	59.8	**0.38 (0.36–0.40)**	**1.16 (1.07–1.25)**
2	134 795/197 163	68.4	**0.55 (0.53–0.58)**	**1.11 (1.06–1.16)**
3	157 704/212 869	74.1	**0.73 (0.70–0.76)**	1.04 (1.00–1.08)
4	164 294/211 798	77.6	**0.89 (0.85–0.93)**	0.97 (0.93–1.00)
5 (least deprived)	167 587/210 538	79.6	1.00	**0.88 (0.83–0.93)**
Health Board (HB)				Residency^*^HB
Ayrshire & Arran	59 385/80 995	73.3	**1.39 (1.25–1.55)**	0.95 (0.90–1.00)
Borders	18 896/24 886	75.9	**1.60 (1.45–1.76)**	**0.92 (0.85–0.99)**
Dumfries & Galloway	20 914/27 382	76.4	**1.64 (1.48–1.81)**	1.07 (0.99–1.15)
Fife	46 822/65 094	71.9	**1.30 (1.17–1.44)**	1.04 (0.99–1.11)
Forth Valley	44 511/61 835	72.0	**1.30 (1.17–1.44)**	0.96 (0.91–1.02)
Grampian	79 486/101 041	78.7	**1.87 (1.69–2.06)**	**1.11 (1.06–1.17)**
Greater Glasgow & Clyde	143 210/215 679	66.4	1.00	**1.25 (1.15–1.36)**
Highland	52 411/68 263	76.8	**1.67 (1.53–1.83)**	1.01 (0.96–1.06)
Lanarkshire	93 394/133 778	69.8	**1.17 (1.05–1.30)**	1.01 (0.96–1.07)
Lothian	102 286/144 108	71.0	**1.24 (1.11–1.37)**	**1.13 (1.07–1.20)**
Orkney	2793/3414	81.8	**2.28 (1.98–2.61)**	**0.76 (0.59–0.98)**
Shetland	5259/6263	84.0	**2.65 (2.33–3.01)**	1.10 (0.91–1.33)
Tayside	62 677/81 771	76.6	**1.66 (1.49–1.85)**	1.05 (1.00–1.11)
Western Isles	5069/6492	78.1	**1.80 (1.61–2.02)**	1.09 (0.92–1.28)

^a^Adjusted for year, age group and the interactions residency^*^SIMD and residency^*^Health Board. For residency^*^SIMD, estimates of the adjusted odds ratio (95% CI) represent the adjusted odds ratio of rural versus urban residency for each SIMD quintile. For residency^*^Health Board, estimates of the adjusted odds ratio (95% CI) represent the adjusted odds ratio (95% CI) of rural versus urban residency for each Health Board. Estimates in bold denote *P* < .05.

### Bowel screening

Of all screening programmes, bowel had the lowest uptake ranging from 53.0% to 66.9% depending on health board, with 56.6% and 62.5% uptake in urban and rural areas, respectively (Table 4). Uptake was lowest among males; those aged 50–54 years and those living in the most deprived areas (SIMD quintile 1). The odds of uptake were 21% lower among males and were higher in all age groups compared to the youngest. Interactions occurred between residency and both SIMD and health board. Among those residing in the most deprived quintile, rural uptake of bowel screening was higher than urban (aOR 1.19, 95% CI 1.17–1.22) with a similar pattern in those living in SIMD 2 and 3. However, uptake was lower among rural residents than urban in the least deprived SIMD quintile. In most health boards, the chance of participation in bowel screening was higher in rural than urban areas. In two health boards, the odds of uptake were lower in rural areas. No differences were seen for two health boards serving exclusively island communities. The bowel cancer screening programme detected 1.17 cancers per 1000 screened (95% CI 1.14–1.21) in urban residents (5089 cancers), with a similar rate in rural areas: 1.15 per 1000 (95% CI 1.09–1.21) (1411 cancers).

**Table 4:** Bowel screening uptake among patients aged 50–74 in Scotland.

Group	Screened/Invited, N	Uptake %	Unadjusted odds ratio (95% CI)	Adjusted odds ratio (95% CI)^a^
Residency				
Urban	4 333 763/7 659 667	56.6	1.00	-
Rural	1 224 935/1 960 226	62.5	**1.28 (1.26–1.30)**	-
Year				
2009/10	407 667/754 055	54.1	1.00	1.00
2010/11	445 435/812 753	54.8	**1.03 (1.02–1.04)**	**1.03 (1.02–1.04)**
2011/12	461 811/841 182	54.9	**1.03 (1.03–1.04)**	**1.03 (1.03–1.04)**
2012/13	468 171/835 805	56.0	**1.08 (1.08–1.09)**	**1.08 (1.07–1.09)**
2013/14	500 533/867 168	57.7	**1.16 (1.15–1.17)**	**1.16 (1.15–1.17)**
2014/15	503 092/866 565	58.1	**1.18 (1.17–1.18)**	**1.18 (1.17–1.19)**
2015/16	509 709/903 114	56.4	**1.10 (1.09–1.11)**	**1.09 (1.08–1.10)**
2016/17	497 726/887 957	56.0	**1.08 (1.08–1.09)**	**1.07 (1.06–1.08)**
2017/18	546 913/933 292	58.6	**1.20 (1.20–1.21)**	**1.19 (1.18–1.20)**
2018/19	599 057/933 626	64.2	**1.52 (1.51–1.53)**	**1.52 (1.51–1.54)**
2019/20	579 048/923 607	62.7	**1.43 (1.42–1.44)**	**1.41 (1.40–1.43)**
2020/21	39 536/60 769	65.1	**1.58 (1.56–1.61)**	**1.57 (1.53–1.61)**
Sex: Female	2 965 222/4 891 124	60.6	1.00	
Male	2 593 476/4 728 769	54.8	**0.79 (0.79–0.79)**	**0.79 (0.79–0.79)**
Age group, years				
50–54	1 414 988/2 828 997	50.0	1.00	1.00
55–59	1 023 535/1 827 065	56.0	**1.27 (1.27–1.28)**	**1.26 (1.25–1.27)**
60–64	1 269 575/2 083 876	60.9	**1.56 (1.55–1.56)**	**1.54 (1.53–1.54)**
65–69	944 278/1 427 170	66.2	**1.95 (1.95–1.96)**	**1.91 (1.90–1.92)**
70–74	906 322/1 452 785	62.4	**1.66 (1.65–1.66)**	**1.63 (1.62–1.64)**
SIMD quintile				Residency^*^SIMD
1 (most deprived)	814 979/1 795 742	45.4	**0.41 (0.40–0.42)**	**1.19 (1.17–1.22)**
2	1 002 618/1 903 850	52.7	**0.55 (0.53–0.56)**	**1.10 (1.09–1.12)**
3	1 201 862/2 028 553	59.2	**0.72 (0.70–0.73)**	**1.09 (1.07–1.10)**
4	1 256 779/1 977 987	63.5	**0.86 (0.84–0.88)**	1.00 (0.99–1.01)
5 (least deprived)	1 282 460/1 913 761	67.0	1.00	**0.93 (0.92–0.95)**
Health Board (HB)				Residency^*^HB
Ayrshire & Arran	428 432/747 548	57.3	**1.16 (1.10–1.21)**	**0.98 (0.96–0.99)**
Borders	149 906/236 726	63.3	**1.49 (1.43–1.55)**	**0.91 (0.88–0.92)**
Dumfries & Galloway	195 972/321 134	61.0	**1.35 (1.29–1.41)**	**1.05 (1.02–1.07)**
Fife	401 210/686 212	58.5	**1.21 (1.16–1.27)**	**1.09 (1.07–1.11)**
Forth Valley	319 195/547 720	58.3	**1.20 (1.15–1.26)**	**1.03 (1.01–1.05)**
Grampian	628 497/992 411	63.3	**1.49 (1.43–1.55)**	**1.14 (1.13–1.16)**
Greater Glasgow & Clyde	1 062 995/1 978 867	53.7	1.00	**1.18 (1.15–1.21)**
Highland	394 867/635 247	62.2	**1.42 (1.36–1.48)**	**1.09 (1.08–1.11)**
Lanarkshire	621 868/1 173 222	53.0	0.97 (0.93–1.02)	**1.05 (1.03–1.07)**
Lothian	795 504/1 390 035	57.2	**1.15 (1.10–1.21)**	**1.13 (1.11–1.15)**
Orkney	27 907/43 847	63.6	**1.51 (1.43–1.59)**	0.97 (0.91–1.03)
Shetland	26 953/40 266	66.9	**1.74 (1.65–1.84)**	1.06 (0.99–1.13)
Tayside	472 345/770 730	61.3	**1.36 (1.30–1.43)**	**1.12 (1.10–1.14)**
Western Isles	33 047/55 928	59.1	**1.24 (1.18–1.32)**	**1.10 (1.04–1.16)**

^a^Adjusted for year, sex, age group and the interactions residency^*^SIMD and residency^*^Health Board. For residency^*^SIMD, estimates of the adjusted odds ratio (95% CI) represent the adjusted odds ratio of rural versus urban residency for each SIMD quintile. For residency^*^Health Board, estimates of the adjusted odds ratio (95% CI) represent the adjusted odds ratio (95% CI) of rural versus urban residency for each Health Board. Estimates in bold denote *P* < .05.

### Drive time

When the multivariable logistic regression models for screening uptake were repeated, including drive time to large settlements instead of rural residency, similar patterns were seen (Supplementary Tables S1, S2). Namely, the association between screening uptake and longer drive time varied by deprivation and in different health board areas across mainland Scotland.

## Discussion

### Main findings of this study

The association between screening uptake and rurality, as measured by population size and accessibility, varied across screening programmes with differing delivery modes. The relationship between rurality and screening uptake also varied in different areas of Scotland and by deprivation. Therefore, the relationship between rural residency and uptake of cancer screening in Scotland was complex, without any obvious rural inequality as suggested in earlier research.

### What is already known on this topic

In a systematic review of cervical screening uptake most included studies (11/18) reported lower cervical screening uptake in rural areas with a higher rural uptake seen in only five studies.^14^ The only UK-based study included examined data from 1998/1999 and found slightly higher screening uptake in rural areas than urban for both 3 year (rural: 69.7%, urban (outside London): 67.9%, urban (London): 65.5%) and 5 year (rural: 86.2%, urban (outside London): 85.5%, urban (London): 80.7%) recall systems.^23^ However, socio-economic circumstances or data from Scotland were not examined.

Of studies (30/32) in a systematic review of studies of rural–urban variations in breast screening uptake; 10 studies (one conducted in the Highland region of Scotland^24^) reported higher rural uptake; 20 studies reported higher urban uptake.^14^ The only other UK study of North Derbyshire, UK found no difference in mammography uptake by rural or urban location.^25^

A recent study examined data from most (3135/3143) US counties and found that the prevalence of colorectal, breast and cervical screening uptake was lower in rural than urban areas, however, for cervical and colorectal screening there were larger differences seen across rural counties than between rural and urban counties.^26^ This subtle variation in the effect of rurality on screening uptake, dependent on the location of the rural area, is consistent with our findings.

### What this study adds

This study provides evidence of the complex interplay between place of residence (whether assessed based on population size or drive time to access services) and uptake of cancer screening in Scotland. For cervical screening, where patients travel to their general practice, the relationship between screening uptake and location varied in just over 25% of health boards for the under 50 years age group and in half of health boards for the 50–64 years group, with no consistent rural disadvantage demonstrated.

Our study found that although mammography uptake varied according to location in just over one third of Scotland’s health boards, the pattern was not consistent. In comparison to urban, rural uptake of breast screening was more likely in the most deprived and least likely in the most affluent areas.

For bowel screening, administered using the postal service, uptake was more likely in rural than urban settings in the most deprived areas. Uptake varied by residency in more than 80% of health boards with most, showing higher bowel screening uptake in rural than urban areas. Although the systematic review found that colorectal cancer screening uptake was higher in rural areas, none of the studies were of UK populations^14^ which means that the screening modality could be different, including colonoscopy or sigmoidoscopy in addition to, or instead of, FOBT and FIT.^14^

It is important to consider why our study found differences in the effects of rurality in cancer screening across different health boards. How cancer screening is organized, accessed and its intersection with regional geography, as well as screening modality, might offer potential explanations. The home-based bowel screening has lower uptake than the clinic-based cervical and breast screening programmes. Barriers to bowel screening participation among women who attended breast and cervical screening in Scotland have been examined^27^ and suggests complex obstacles. Reasons for not returning kits included: fear of colorectal cancer treatment; a belief that colorectal cancer symptoms were easier to self-detect than those of breast or cervical cancer; the home-based screening test was viewed as easy to forget or delay; worries about incorrectly completing the test without health care professional support and feelings of disgust and embarrassment about the self-test.^27^ The pattern of bowel screening uptake was consistent across most health boards with a higher uptake in rural compared to urban areas and could relate to screening modality as those eligible receive FIT kits direct to their home.

Scotland’s rural communities contain fewer geographical concentrations of deprivation than poverty rates suggest.^28^ According to the SIMD classification, fewer people in rural than urban areas are living in the most deprived areas.^29^ However, in rural areas, the SIMD a spatially-based classification, designed to identify geographical clusters of deprived households, does not capture the extent of disadvantaged households who live in sparsely populated, geographically dispersed communities.^30^ Concentrations of deprived households are more easily identified in population centres, commonly where social housing and core services, including general practices are located. The heterogeneous nature of rural communities is perhaps more likely to impact the uptake of cervical and breast than bowel screening. People who live outside a small town will have the furthest to travel to access screening, whether it’s provided at their general practice or a mobile screening unit. For those employed, their workplace may be at a distance from home and not necessarily near where cervical or breast screening is accessed, making it difficult to attend. Practice list size and location could influence screening uptake because these practice characteristics are associated with ease of booking cervical screening appointments. Examination of practice list sizes^31^ (Supplementary Table S3) suggests that health boards where rural uptake of cervical screening is higher than urban tend to be comprised of practices with smaller lists.

Uptake of mammography requires travel to a breast screening centre or visiting mobile unit. The associated travel burden due to distance, nature of the journey (by road, ferry or air), and public transport availability is likely to vary across different rural areas and in turn, influence screening attendance. However, in most Scottish health boards, there were no rural–urban variations or rural uptake of mammography was higher, which suggests that mobile screening units currently successfully mitigate travel burden for many patients.

### Limitations of this study

Our study was based on aggregate rather than individual-level data, and thus we must avoid ecological fallacy.^32^ That said community/area level factors are important for example in stage at breast cancer diagnosis^33^ in addition to individual-level risk factors and the context of health care delivery.^34^ Data about ethnicity was not available. A small proportion of Scotland’s population is from ethnic minority groups with urban, mainly city populations being more diverse than rural^35^ so any potential influence of ethnicity would likely impact screening uptake more in urban than rural areas.

## Conclusion

There is not a pan-Scotland rural–urban gradient in cancer screening uptake. Indeed, geographical influences on screening may act more regionally, at the level of local topography and service organisation. The current structure of 14 regional health boards in Scotland may support attempts to increase screening uptake through targeted and tailored local public health initiatives rather than a single national policy.

## Supplementary Material

fdag018_Supplementary_materials

## Data Availability

Aggregate cancer screening data were obtained from Public Health Scotland (approval reference DP2122–0245 IR2021–00460). Permission to access the cancer screening data to be sought from Public Health Scotland.
