# Supplementary material for: The influence of rural residency on the uptake of screening for breast, cervical, and colorectal cancers in Scotland
Source: J Public Health (Oxf). 2026 Feb 27;48(2):467–76. doi: 10.1093/pubmed/fdag018 (PMC13223569; doi:10.1093/pubmed/fdag018)
Supplement: fdag018_Supplementary_materials [file fdag018_supplementary_materials.docx]

**Supplementary material for “The influence of rural residency on the uptake of screening for breast, cervical and colorectal cancers in Scotland”**

**Table S1. Cancer screening uptake according to drive time in mainland Scotland.***

|  | Drive time, minutes | |
| --- | --- | --- |
|  | ≤30 | >30 |
| Cancer Screening Programme | % (Screened/Invited, N) | % (Screened/Invited, N) |
| Bowel | 57.2 (4889054/8549889) | 62.5 (581737/929963) |
| Breast | 71.5 (651190/910191) | 76.9 (72802/94641) |
| Cervical (3.5 years) | 68.0 (1464333/2154620) | 73.0 (981234/1343064) |
| Cervical (5.5 years) | 76.2 (935277/1226681) | 74.9(620062/827650) |

*Excludes cancer screening uptake data from Orkney, Shetland and Western Isles health board areas.

**Table S2. Cancer screening uptake multivariable logistic models incorporating drive time.***

| Group | Cancer screening uptake, adjusted odds ratio (95% CI) | | | |
| --- | --- | --- | --- | --- |
|  | Bowel | Breast | Cervical (3.5 years) | Cervical (5.5 years) |
| Year |  |  |  |  |
| 2009/10 | 1.00 |  |  |  |
| 2010/11 | **1.03 (1.02, 1.04)** |  |  |  |
| 2011/12 | **1.04 (1.03, 1.04)** |  |  |  |
| 2012/13 | **1.09 (1.08, 1.10)** |  |  |  |
| 2013/14 | **1.16 (1.15, 1.17)** |  |  |  |
| 2014/15 | **1.18 (1.17, 1.19)** |  |  |  |
| 2015/16 | **1.09 (1.08, 1.10)** |  |  |  |
| 2016/17 | **1.08 (1.07, 1.09)** | 1.00 | 1.00 | 1.00 |
| 2017/18 | **1.19 (1.18, 1.20)** | **0.94 (0.93, 0.96)** | 0.98 (0.96, 1.00) | **0.97 (0.96, 0.98)** |
| 2018/19 | **1.53 (1.51, 1.54)** | **1.14 (1.12, 1.16)** | 1.00 (0.98, 1.02) | **0.98 (0.97, 0.99)** |
| 2019/20 | **1.42 (1.40, 1.43)** | 1.01 (0.99, 1.02) | **0.90 (0.88, 0.92)** | **0.93 (0.92, 0.94)** |
| 2020/21 | **1.57 (1.53, 1.61)** |  |  |  |
|  |  |  |  |  |
|  |  |  |  |  |
| Sex: Female | 1.00 |  |  |  |
| Male | **0.79 (0.79, 0.79)** |  |  |  |
|  |  |  |  |  |
|  |  |  |  |  |
| Age group, years |  |  |  |  |
| 25-29 |  |  | 1.00 |  |
| 30-34 |  |  | **1.37 (1.34, 1.40)** |  |
| 35-39 |  |  | **1.59 (1.56, 1.63)** |  |
| 40-44 |  |  | **1.74 (1.70, 1.78)** |  |
| 45-49 |  |  | **1.76 (1.72, 1.80)** |  |
| 50-54 | 1.00 | 1.00 |  | 1.00 |
| 55-59 | **1.26 (1.25, 1.27)** | **1.07 (1.05, 1.08)** |  | **0.82 (0.81, 0.82)** |
| 60-64 | **1.54 (1.53, 1.55)** | **1.14 (1.12, 1.16)** |  | **0.64 (0.64, 0.65)** |
| 65-69 | **1.91 (1.90, 1.93)** | **1.13 (1.11, 1.15)** |  |  |
| 70-74 | **1.63 (1.62, 1.64)** |  |  |  |
|  |  |  |  |  |
|  |  |  |  |  |
| SIMD quintile | Drive time*SIMD | Drive time*SIMD | Drive time*SIMD | Drive time*SIMD |
| 1 (most deprived) | **1.18 (1.08, 1.28)** | 1.22 (0.98, 1.53) | **0.87 (0.84, 0.90)** | 0.98 (0.96, 1.00) |
| 2 | **1.18 (1.09, 1.28)** | 1.13 (0.91, 1.40) | **1.04 (1.01, 1.08)** | 0.99 (0.97, 1.01) |
| 3 | **1.12 (1.03, 1.21)** | 0.98 (0.80, 1.22) | **1.22 (1.18, 1.27)** | 0.99 (0.97, 1.01) |
| 4 | 1.04 (0.96, 1.13) | 0.95 (0.77, 1.18) | **1.16 (1.12, 1.21)** | 1.01 (0.99, 1.03) |
| 5 (least deprived) | 1.01 (0.93, 1.10) | 0.90 (0.72, 1.14) | **1.33 (1.28, 1.39)** | **1.09 (1.07, 1.12)** |
|  |  |  |  |  |
|  |  |  |  |  |
| Health Board (HB) | Drive time*HB | Drive time*HB | Drive time*HB | Drive time*HB |
| Ayrshire and Arran | **1.07 (1.05, 1.10)** | **1.09 (1.01, 1.16)** | 1.01 (0.94, 1.08) | 0.99 (0.96, 1.02) |
| Borders | **1.04 (1.01, 1.08)** | 1.07 (0.96, 1.19) | 1.12 (0.99, 1.26) | **1.23 (1.16, 1.31)** |
| Dumfries and Galloway | 1.03 (1.00, 1.05) | 1.00 (0.92, 1.09) | 1.02 (0.92, 1.14) | 1.02 (0.97, 1.07) |
| Fife | 1.52 (1.00, 2.30) | 0.95 (0.25, 3.63) | 0.99 (0.93, 1.06) | 1.02 (0.99, 1.05) |
| Forth Valley | **1.10 (1.05, 1.16)** | 1.05 (0.92, 1.20) | **0.92 (0.85, 0.99)** | 0.96 (0.93, 1.00) |
| Grampian | **1.17 (1.15, 1.19)** | **1.17 (1.11, 1.25)** | **1.15 (1.09, 1.21)** | 1.01 (0.98, 1.04) |
| Greater Glasgow and Clyde | 0.97 (0.44, 2.12) | 0.69 (0.11, 4.49) | **1.59 (1.52, 1.66)** | **0.97 (0.95, 0.99)** |
| Highland | **1.05 (1.03, 1.06)** | 1.03 (0.97, 1.08) | **0.92 (0.87, 0.99)** | 0.98 (0.95, 1.01) |
| Lanarkshire | 1.01 (0.95, 1.07) | **1.27 (1.03, 1.56)** | 0.99 (0.94, 1.04) | **0.92 (0.90, 0.94)** |
| Lothian | **1.20 (1.16, 1.24)** | **1.16 (1.04, 1.30)** | **1.65 (1.59, 1.72)** | **1.05 (1.03, 1.08)** |
| Tayside | **1.05 (1.03, 1.08)** | 0.98 (0.91, 1.05) | **1.12 (1.06, 1.19)** | 1.01 (0.98, 1.04) |

* For the interaction terms of SIMD with drive time, estimates are the adjusted odds ratio (95% CI) of drive time>30 minutes compared to drive time ≤30 minutes for each SIMD quintile. For the interaction terms of HB with Drivetime, estimates are the adjusted odds ratio (95% CI) of drivetime >30 minutes compared to drive time ≤ 30 for each Health Board. Estimates in bold denote p<0.05.

**Table S3. General practice list sizes by Health Board in Scotland.**

| Health Board | Total of all general practice list sizes | Median list size (interquartile range) | Number of practices in a rural location (%) | Number of practices in an urban location (%) |
| --- | --- | --- | --- | --- |
| Ayrshire & Arran | 387,047 | 7119 (5944) | 10 (18.5) | 44 (81.5) |
| Borders | 120,764 | 4906 (3164) | 9 (39.1) | 14 (60.9) |
| Dumfries & Galloway | 155,589 | 4463 (2938) | 14 (43.7) | 18 (56.3) |
| Fife | 390,539 | 7312 (3866) | 6 (11.3) | 47 (88.7) |
| Forth Valley | 325,061 | 6091 (4394) | 12 (24.0) | 38 (76.0) |
| Grampian | 604,967 | 8348 (5787) | 21 (30.4) | 48 (69.6) |
| Greater Glasgow & Clyde | 1,339,333 | 5142 (4367) | 2 (0.9) | 230 (91.1) |
| Highland | 334,092 | 2468 (4417) | 62 (65.3) | 33 (34.7) |
| Lanarkshire | 695,810 | 6406 (4918) | 5 (5.0) | 95 (95.0) |
| Lothian | 1,008,183 | 8324 (4080) | 7 (5.9) | 112 (94.1) |
| Orkney | 22,217 | 2871 (1738) | 6 (85.7) | 1 (14.3) |
| Shetland | 22,994 | 1906 (1743) | 8 (88.9) | 1 (10.1) |
| Tayside | 434,730 | 6957 (4614) | 11 (17.5) | 52 (82.5) |
| Western Isles | 27,098 | 1397 (4040) | 7 (77.8) | 2 (22.2) |
